# Quantitative optical coherence tomography angiography biomarkers for neovascular age-related macular degeneration in remission

**DOI:** 10.1371/journal.pone.0205513

**Published:** 2018-10-09

**Authors:** Florence Coscas, Diogo Cabral, Telmo Pereira, Carlos Geraldes, Hemaxi Narotamo, Alexandra Miere, Marco Lupidi, Alexandre Sellam, Ana Papoila, Gabriel Coscas, Eric Souied

**Affiliations:** 1 Centre Ophtalmologique de l’Odéon, Saint Germain, Paris, France; 2 Department of Ophthalmology, Centre Hospitalier Intercommunal de Creteil University Paris Est Creteil XIl, Créteil, France; 3 NOVA Medical School, Faculdade de Ciências Médicas, Universidade Nova de Lisboa, Lisbon, Portugal; 4 CEDOC, Chronic Diseases Research Center, Universidade Nova de Lisboa, Lisbon, Portugal; 5 Instituto de Oftalmologia Dr. Gama Pinto, Lisbon, Portugal; 6 CEAUL, Centro de Estatística e Aplicações da Universidade de Lisboa, Lisbon, Portugal; 7 Department of Biomedical and Surgical Sciences, Section of Ophthalmology, University of Perugia, S. Maria della Misericordia Hospital, Perugia, Italy; 8 Quinze-Vingts National Eye Hospital, Faculté de Médecine Sorbonne Université, Paris, France; Massachusetts Eye & Ear Infirmary, Harvard Medical School, UNITED STATES

## Abstract

**Purpose:**

To characterize quantitative optical coherence tomography angiography (OCT-A) parameters in active neovascular age-related macular degeneration (nAMD) patients under treatment and remission nAMD patients.

**Design:**

Retrospective, cross-sectional study.

**Participants:**

One hundred and four patients of whom 72 were in Group 1 (active nAMD) and 32 in Group 2 (remission nAMD) based on SD-OCT (Spectral Domain OCT) qualitative morphology.

**Methods:**

This study was conducted at the *Centre Ophtalmologique de l’Odeon* between June 2016 and December 2017. Eyes were analyzed using SD-OCT and high-speed (100 000 A-scans/second) 1050-nm wavelength swept-source OCT-A. Speckle noise removal and choroidal neovascularization (CNV) blood flow delineation were automatically performed. Quantitative parameters analyzed included blood flow area (Area), vessel density, fractal dimension (FD) and lacunarity. OCT-A image algorithms and graphical user interfaces were built as a unified tool in Matlab coding language. Generalized Additive Models were used to study the association between OCT-A parameters and nAMD remission on structural OCT. The models’ performance was assessed by the Akaike Information Criterion (AIC), Brier Score and by the area under the receiver operating characteristic curve (AUC). A p value of ≤ 0.05 was considered as statistically significant.

**Results:**

Area, vessel density and FD were different (p<0.001) in the two groups. Regarding the association with CNV activity, Area alone had the highest AUC (AUC = 0.85; 95%CI: 0.77–0.93) followed by FD (AUC = 0.80; 95%CI: 0.71–0.88). Again, Area obtained the best values followed by FD in the AIC and Brier Score evaluations. The multivariate model that included both these variables attained the best performance considering all assessment criteria.

**Conclusions:**

Blood flow characteristics on OCT-A may be associated with exudative signs on structural OCT. In the future, analyses of OCT-A quantitative parameters could potentially help evaluate CNV activity status and to develop personalized treatment and follow-up cycles.

## Introduction

Neovascular age-related macular degeneration (nAMD) is one of the leading causes of visual impairment in western countries.[1] The basic disease process in nAMD is choroidal neovascularization (CNV) which is characterized by the growth of blood vessels through the Bruch’s membrane and is associated with invasion of immune and non-immune cells. Actually, CNV is regarded as an attempt to compensate for reduced choriocapillaris as this can be compromised in patients with late age-related macular degeneration.[2]

Fluorescein angiography (FA) is considered as the gold standard in the diagnosis and classification of CNV type 1 (under the retinal pigment epithelium) or type 2 (extension into the subretinal space).[3] However, as a dye based method, FA imaging is influenced by leakage and is not suitable to study blood flow morphological features.

Optical Coherence Tomography-angiography (OCT-A) is a non-dye diagnostic technique that has been evaluated as an alternative or a complement to FA and indocyanine green angiography (ICG-A).[4] Comparative studies have demonstrated that OCT-A can detect CNV blood flow with the same sensitivity as FA[5,6] and is able to show the area of type 1 CNV more precisely than ICG-A.[7]

Previous studies using OCT-A have demonstrated that CNV blood flow was clearly observed in eyes with active and remission phases of nAMD while eyes undergoing CNV treatment showed different morphological features in neovascular networks varying from an homogeneous, tiny branching network to an heterogeneous dead tree appearance.[6] These studies demonstrated that the appearance of CNV blood flow may have its own importance.

The identification of biomarkers for lesion activity has become a hot topic in retinal clinical research. The currently available and reproducible[8] methods for the quantification of CNV characteristics rely on semiautomatic software to define the CNV area and its boundaries, enabling the CNV to be followed up along the course of the disease.[9] Recent studies have demonstrated that the application of fractal dimension (FD) analysis may be useful in differentiating blood flow in the quiescent, active and remission phases of the disease.[10] Other studies have suggested that lacunarity (LAC) or junction density could be an objective way of following and managing CNV.[11] However, the predictive performance of these new OCT-A biomarkers are yet to be evaluated.

The aim of our study was to characterize quantitative OCT-A parameters in active nAMD patients under treatment and remission nAMD patients.

## Material and methods

### Study design

Retrospective, cross-sectional review analysis of anonymous imaging data acquired during usual clinical practice from consecutive patients diagnosed with nAMD. This study had Institutional Review Board approval from Paris Est University and was conducted in accordance with the tenets of the Declaration of Helsinki (1964) and the French legislation. All enrolled patients gave their written consent at the time of recruitment.

### Participants

The patients treated at the *Centre Ophtalmologique de l’Odeon* between June 2016 and December 2017 were included in the active group (Group 1) if they met all the following inclusion criteria: 1) diagnosis of nAMD by multimodal imaging (Structural OCT, FA and ICG-A); 2) Type 1, Type 2 CNV; 3) at least one IVI in the last 3 months); 4) exudative structural OCT signs (intra or subretinal fluid, presence of subretinal pigment epithelial detachment in type 1 or pre-epithelial hyper reflectivity in type 2 cases).

Patients included in the remission, inactive group (Group 2) met all the following criteria: 1) initial type 1, type 2 or mixed CNV; 2) absence of sign of activity (intra or subretinal fluid) and the presence of hyper reflective fibrosis on structural OCT 3) the most recent IVI more than 6 months ago.

We excluded Type 3 neovascularization, polypoidal choroidal vasculopathy (PCV) or other retinal confounding diseases complicated with secondary CNV; poor quality images on OCT-A (signal strength index lower than 80); neovascular network that exceeded the 4.5x4.5 mm scanning area used on OCT-A; multiple lesions of CNV; central geographic atrophy and images with motion artifacts.

The diagnosis of CNV subtype was made on initial multimodal imaging including FA, ICG-A and structural OCT scans. CNV classification was independently reviewed by two experienced examiners (FC, GC). Active type 1 CNV was defined as late hyperfluorescent leakage and pin points on FA, vascularized pigment epithelium detachment (PED) on ICG-A and PED, associated with intraretinal and/or subretinal fluid on structural OCT. Active type 2 CNV was defined as an area of well-defined early hyperfluorescence and leakage on FA and localized pre-epithelial hyperreflectivity with fluids on structural OCT.[12–14]

The treatment protocol was *pro re nata* and included retreatment if there was any evidence of disease activity on structural OCT: intra or subretinal fluid and increase of central macular thickness.[15,16]

### Data Sources

Comprehensive ophthalmic examination of the study patients including best corrected visual acuity (BCVA) using the ETDRS (Early Treatment of Diabetic Retinopathy Study) visual chart, retinal photography of the fundus, structural OCT and swept-source OCT-A. Evaluation of the patients’ baseline characteristics included age, gender, central macular thickness (CMT), disease duration and number of previous anti-VEGF IVIs.

### Image acquisition and analysis

Enhanced Depth Imaging (EDI) Spectral domain OCT with automated volume mode of 49 B-scans and macular 30 degrees centered on the fovea was performed using Heyex v6.9a (Spectralis OCT, Heidelberg Engineering Inc., Heidelberg, Germany). Swept-source optical coherence tomography angiography images 4.5x4.5mm sections centered on the fovea were obtained with the DRI OCT Triton (Topcon, Tokyo, Japan). IMAGEnet 6 version 1.21 was used to evaluate outer retinal layers, between the outer plexiform layer and the Bruch’s membrane, for blood flow abnormalities suggestive of CNV.[17–19] Projection artifacts were subtracted automatically using the “reverse shadowing” effect of the retinal vessels available in this software.[20] Images were exported as Tagged Image File Format (TIFF) for analysis.

### Quantitative variables

**Fig 1:**
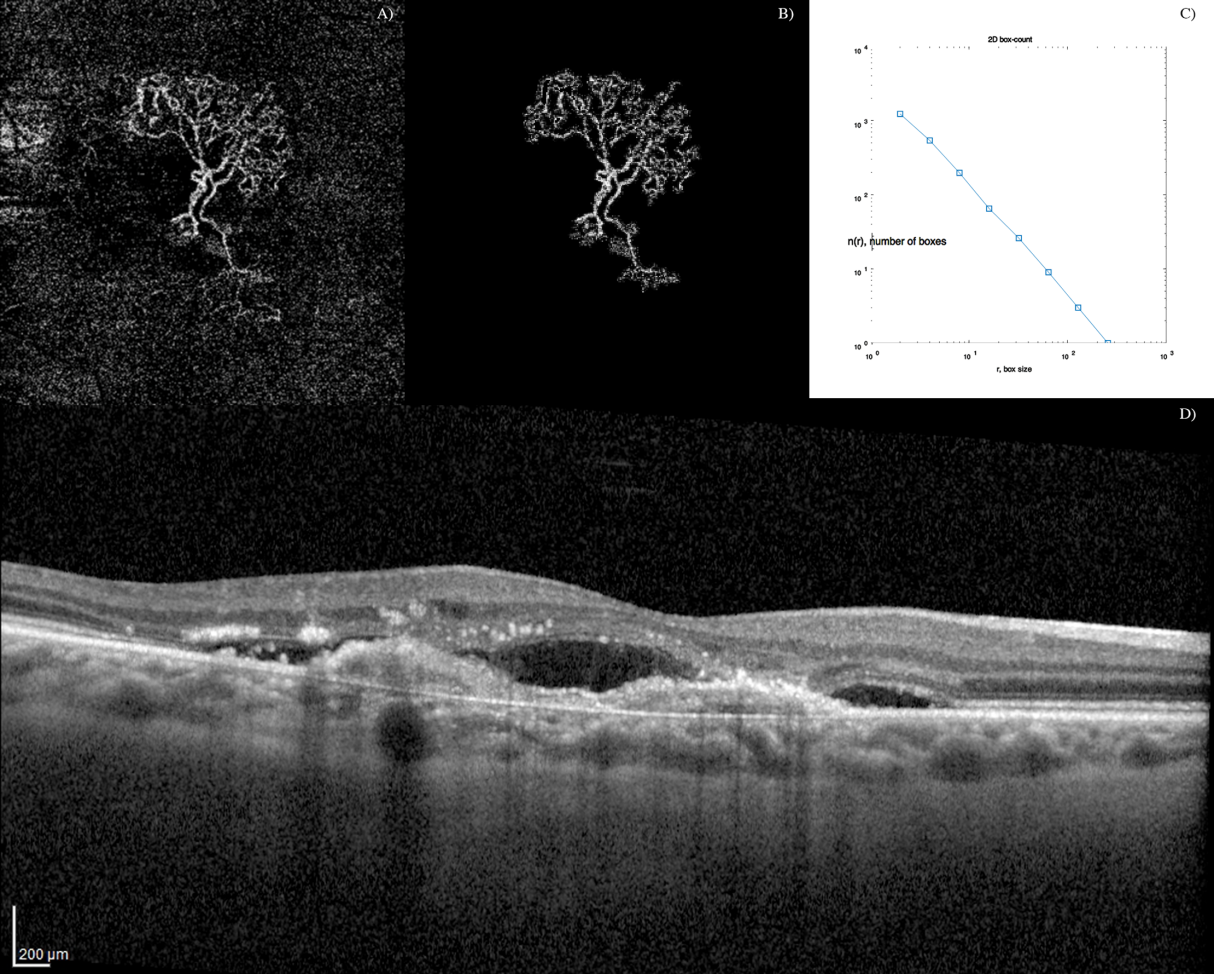
Optical coherence tomography angiography (OCTA) images showing the filtering schema on a dense net hypersignal of active choroidal neovascularization. A) OCTA outer retina layers blood flow image. B) Speckle noise removal and automatic blood flow delineation. C) Graphic with the output of the box-counting method: N (the number of boxes needed to cover the set) as a function of R (the size of the boxes); as the set is a fractal, we can observe a power-law relationship [N = N0 * R^(-DF)], being DF the fractal dimension (Kolmogorov capacity). D) Corresponding optical coherence tomography (OCT) B-scan showing a active type 1 CNV with sub-retinal fluid.

**Fig 2:**
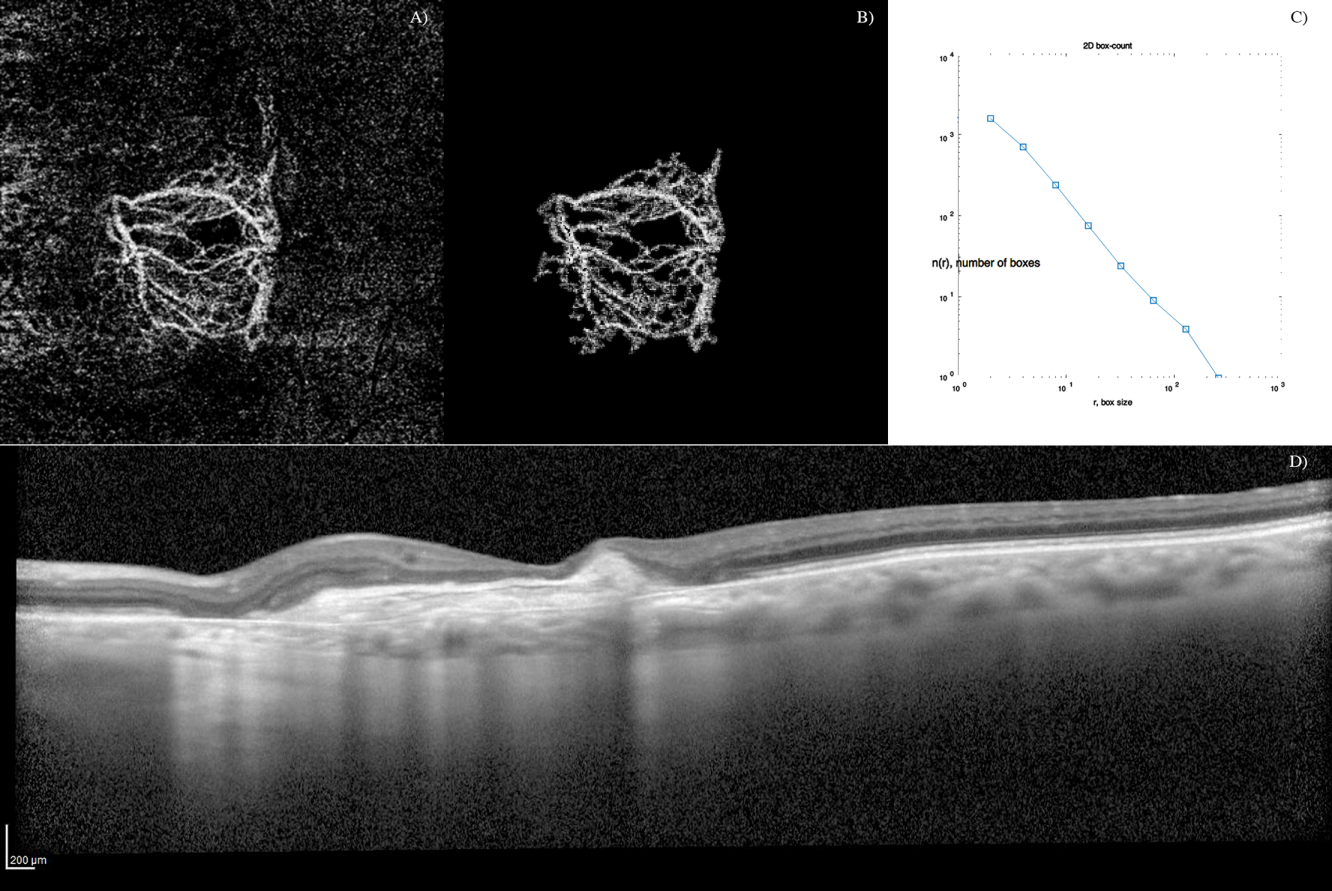
Optical coherence tomography angiography (OCTA) images showing the filtering schema on a loose net hypersignal of inactive choroidal neovascularization. A) OCT-A outer retina layers blood flow image. B) Speckle noise removal and automatic blood flow delineation. C) Graphic with the output of the box-counting method: N (the number of boxes needed to cover the set) as a function of R (the size of the boxes); as the set is a fractal, we can observe a power-law relationship [N = N0 * R^(-DF)], being DF the fractal dimension (Kolmogorov capacity). D) Corresponding optical coherence tomography (OCT) B-scan showing a inactive type 1 CNV with sub-retinal fibrosis.

OCT-A image analysis was done using a custom graphical user interface built in MATLAB (v. r2018a) coding language. Images were binarized using the phansalkar local threshold method (rolling ball of 15 pixels).[21] Filtering of speckle noise was achieved by using median filter of radius 2 pixels. Small non-connected pixels (8-connected)—smaller than 10 pixels–were removed. The density map was computed, and the highest density zone was identified. A custom region growing algorithm using region mean threshold ratios as stop condition was used to determine the final CNV shape. Then, blood flow was detected through mathematical morphology analysis (Figs 1 and 2). Quantitative OCT-A analyses of blood flow area, aspect ratio, vessel density, FD and LAC were issued using a graphical interface.

Aspect ratio was defined as the ratio between the large and the small axis of the CNV, and vessel density as the percentage of the CNV area occupied by vessels. The box counting method at multiple origins was applied to the image of the binary skeleton to estimate FD[22] and LAC[23] of the vascular network, which are well-known global indices of morphological complexity and structural nonuniformity, respectively.[24] Box sizes followed the power of 2 series until a box of half image pixel size was reached. The results were automatically exported into a comma separated file for further analysis.

### Statistical analysis

We performed an exploratory study of demographic, clinical, and structural OCT/OCTA measurements (Area, LAC, density, FD, aspect ratio, and CMT). Continuous variables were presented as mean and standard deviation (SD) or median and interquartile range (25th percentile - 75th percentile) and range ([min, max]), as appropriate. We analyzed the association between each of these variables and the binary dependent variable CNV status (Active nAMD vs Remission nAMD). Generalized Additive Models[25] (GAMs) for binary response were used for this purpose and partial functions that translate the referred associations were obtained using splines.

Using the baseline GAM model (with CNV status as outcome and only the covariate area), three multivariable models were constructed adding each one of the remaining covariates (density, FD and LAC) at a time. The goal of this analysis was to quantify the added contribution to the performance of the baseline model by each of these biomarkers regarding the trade-off between the goodness of fit of the model and its complexity measured by the Akaike Information Criterion (AIC), the accuracy of probability estimates measured by the Brier Score, and their discriminative ability to distinguish between active and remission nAMD measured by the area under the receiver operating characteristic (ROC) curve (AUC). Lower values of AIC and of Brier Score indicate a better goodness of fit while higher AUC values indicate better discriminative ability. A p value of ≤ 0.05 was considered as statistically significant. All statistical analyses were performed in R programme.[26]

## Results

### Participants

One hundred and forty patients with nAMD were evaluated. From this cohort, 104 patients fulfilled image quality inclusion criteria and were included in the study: 72 patients were under treatment with anti-VEGF IVI (Group 1) and 32 patients had inactive CNV under remission (Group 2). The overall mean age was 81.1 (7.4) years and 40 (38.5%) study patients were female.

### Descriptive data by group

Description of patients regarding demographic and clinical characteristics, and structural OCTA measurements, by group, is presented in Table 1.

**Table 1:** Demographic and structural and angiography optical coherence tomography characteristics by group.

	Group 1 (Active nAMD)	Group 2 (Remission nAMD)	p-value*
**Age (years)**	81.1 (7.6)	81.1 (7.0)	0.963
**Disease duration (months)**	52.8 (33.6) [8, 125]	189.8 (82.1) [36, 358]	<0.001
**Number of IVI**	17.0 (11.0–27.3) [5, 69]	12.0 (9.0–14.3) [5, 27]	0.003
**VA (ETDRS letters)**	65.0 (49.0–75.0) [13.0, 89.0]	44.0 (31.0–55.0) [3.0, 83.0]	<0.001
**FD**	1.44 (0.09) [1.24, 1.66]	1.50 (0.04) [1.40, 1.58]	<0.001
**LAC**	0.38 (0.06) [0.27, 0.53]	0.39 (0.36–0.42) [0.32, 0.47]	0.195
**Area (mm**^**2**^**)**	1.94 (0.76–3.30) [0.12, 13.03]	5.82 (3.72–8.20) [1.17, 12.82]	<0.001
**Density (%)**	0.49 (0.41–0.56) [0.26, 0.87]	0.39 (0.36–0.45) [0.26, 0.60]	<0.001
**Aspect ratio**	0.70 (0.13) [0.40, 0.93]	0.74 (0.14) [0.46, 0.94]	0.093
**CMT (μm)**	301.5 (257.8–372.3) [185.0, 854.0]	260.0 (214.8–308.5) [130.0, 451.0]	<0.001

Continuous variables were presented as mean and standard deviation (SD) or median and interquartile range (25^th^ percentile - 75^th^ percentile) and range ([min, max]), as appropriate. IVI: Intra vitreal injection–. FD—Fractal dimension. LAC–Lacunarity. nAMD: neovascular Age Related Macular Degeneration. CMT—Central Macular Thickness. VA–Visual Acuity.

*Obtained by chi-squared and Mann–Whitney non parametric tests.

In the active group, mean age was 81.1 (7.6) years with 20 (19.2%) female patients. The median visual acuity at baseline was 65.0 (49.0–75.0) letters, and the median CMT was 301.5 (257.8–372.3) **μ**m. Regarding CNV subtypes, type 1 was expressed in 80.3% (57/71) of the patients and type 2 in 21.1% (15/71).

The median number of intravitreal anti-VEGF treatments received before enrollment was 17.0 (11.0–27.3). There was no association between the number of anti-VEGF treatments and FD (p = 0.766)

In the remission group, the median age was 81.1 (7.0), with 20 (19.2%) female patients. The median visual acuity at baseline was 44.0 (31.0–55.0) letters, and the median CMT was 260.0 (214.8–308.5) **μ**m. Regarding nAMD subtypes, type 1 was expressed in 87.1% (27/31) of the patients and type 2 in 16.1% (5/31). The median number of intravitreal anti-VEGF received before enrollment was 12.0 (9.0–14.3).

There was no significant difference between the two groups regarding age (p = 0.963), and CNV classification (p = 0.724). However, gender (p < 0.001), CMT (p < 0.001) and visual acuity (p < 0.001) were significantly different between the two study groups.

### OCT/OCTA measurements

The median area of the lesion was 1.94 (0.76–3.30) mm^2^ in active group and 5.82 (3.72–8.20) mm^2^ in the remission group.

The median vessel density of the lesion was 0.49 (0.41–0.56) % in the active group and 0.39 (0.36–0.45) % in the remission group.

The mean aspect ratio in active group was 0.70 (0.13) and 0.74 (0.14) in the remission group.

We found a significant (p<0.001) difference between the two study groups regarding lesion area and vessel density but not in aspect-ratio (p = 0.093). The mean FD in the active group was significantly (p<0.001) lower than that in the remission group while the median LAC was similar to that in the remission group (p = 0.195) (Table 1).

### Models’ results

The performance analyses of the models is summarized in Table 2. Regarding the performance of the univariate models (with only one of the variables: area, density, LAC or FD), area (AUC = 0.85, 95%CI: 0.77–0.93, AIC = 97.1 and Brier Score = 0.1417) and LAC (AUC = 0.60, 95%CI: 0.48–0.72, AIC = 126.1 and Brier Score = 0.1982) attained the best and the worst results respectively. In the multivariate models, results showed that when adding each of the remaining OCT-A parameters to area, only FD led to an increase in the discriminative ability of the models. Effectively, the model including area and FD (referred to as the Area + FD model) not only had the best discriminative ability but also the best AIC and Brier Scores. In this last model, the existence of a potential collinearity problem was not confirmed since the introduction of FD in the model did not significantly change the standard error of Area´s regression coefficient.

**Table 2:** Models’ performance analysis.

	AUC (95% CI)	AIC	Brier’s Score
**Dens**	0.75 (0.66, 0.85)	113.2	0.1792
**LAC**	0.65 (0.54, 0.76)	126.1	0.1982
**FD**	0.80 (0.71, 0.88)	106.1	0.1629
**Area**	0.85 (0.77, 0.93)	97.1	0.1417
**Area+Dens**	0.85 (0.77, 0.93)	99.0	0.1420
**Area+LAC**	0.85 (0.78, 0.93)	98.4	0.1397
**Area+FD**	0.91 (0.85, 0.97)	84.4	0.1138

Dens–Vessel Density. LAC–Lacunarity. FD–Fractal dimension. AUC–area under the Receiver Operating Characteristic Curve. CI–Confidence Interval, AIC -Akaike information criterion.

**Fig 3:**
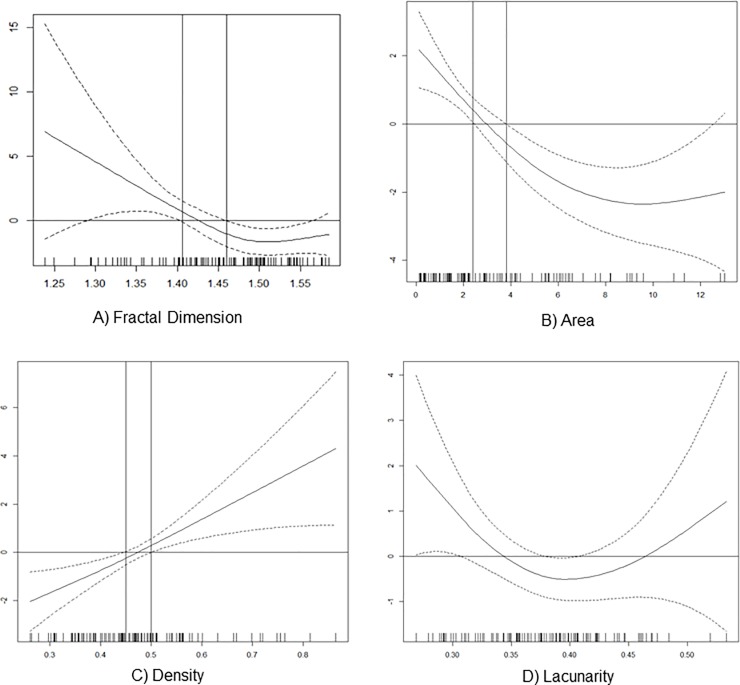
Composite with partial functions showing the association between OCTA quantitative parameters and choroidal neovascularization (CNV) status. A) Association between fractal dimension and the odds of CNV status—higher odds of active CNV correspond to values lower than 1.41, and more protection is related to values higher than 1.46. B) Association between CNV area and the odds of CNV status—higher odds of active CNV is related to values lower than 2.4 and values higher than 3.8 correspond to protection C) Association between vessel density and the odds of CNV status—values below 0.44 correspond to protection and above 0.50 to higher odds of active CNV. D) Association between lacunarity and the odds of CNV status—cutoff values were not obtained as zero value is contained in all the confidence intervals.

Additionally, for each OCTA parameter, a plot that represents a GAM partial function showing the association between the OCTA parameter and the odds of CNV status, was obtained. These plots are shown in Fig 3. Also the cut-off points, with clinical usefulness for each OCTA parameter, were included, with the exception of Lacunarity as zero value is contained in all the confidence intervals as depicted in corresponding partial function plot. In the case of FD, higher odds of active CNV correspond to values lower than 1.41, and more protection is related to values higher than 1.46. In the interval (1.41–1.46), as the 95% confidence intervals contain the zero value, no statistically significant association between FD and CNV status is identified, and accordingly this interval may be viewed as a gray zone. Regarding CNV vessel density, the gray zone lies in the interval (0.44–0.50). Values below 0.44 correspond to protection and above 0.50 to higher odds of active CNV. In the case of CNV area, higher odds of active CNV is related to values lower than 2.4 mm^2^. The gray zone is located in the interval (2.4–3.8). Values higher than 3.8 mm^2^ correspond to protection.

## Discussion

In this study, we applied statistical models based on OCT-A quantitative analysis to associate CNV remission on structural OCT with OCT-A features. We found that the combination of blood flow area and FD model attained the best performance considering all assessment criteria.

OCT-A enables high-resolution visualization of the CNV microvascular network. One of our significant results was the high performance of Area (AUC = 0.85) in the discrimination between active and remission nAMD groups. Its measurement is a readily available and well-studied OCT-A parameter. Comparative studies with ICG-A have shown that as OCTA is not influenced by the phenomenon of leakage, it is able to show the minimal CNV surface.[7] Reproducibility studies have shown that OCTA provides reproducible imaging for accurate evaluation of the CNV size.[8] The growth of neovascular lesions in nAMD has been acknowledged since the MARINA study where it was observed that eyes treated monthly with anti–VEGFs experienced growth of their neovascular lesions after 1 year.[27–29] Therefore, as the duration of the disease in the remission group was longer, it is not surprising that this parameter was a strong discriminative factor. Nevertheless, the model may be further improved if Area is modeled together with FD (better results are achieved in all criteria). Adding Density or LAC does not improve the model.

Surrogate parameters for vascular changes during anti-angiogenic treatments were previously studied in the chick chorioallantoic membrane.[30] Morphological complexity (FD) and structural nonuniformity (LAC) were evaluated using the box-counting method.[24]

FD is a statistical descriptor of space-filling patterns and has a value between 0 and 2 (higher values indicating increased pattern complexity). Al-Sheik *et al* have previously studied the FD of neovascular networks and established a correlation between the branching pattern and complexity assessment by FD analysis.[31] They reported a higher mean FD in 10 active CNV networks compared with 21 inactive networks. Also, the FD value was reduced after treatment with anti-VEGFs suggesting that the pattern of the CNV lesion might be less complex due to the attenuation and pruning of small-caliber vessels.[31] We studied a large cohort of consecutive patients from daily clinical practice and observed that blood flow area was statistically larger in inactive networks compared with active networks. This result is discrepant with the higher mean FD in active CNVs reported by Al-Sheik et al. One explanation could be due to a confounder effect of the number of IVIs between study groups, that we have eliminated by demonstrating no association between the number of IVIs and FD value. Another possible explanation could be the inclusion of CNVs with different phases of arteriogenesis between the two studies. As shown by previous qualitative assessments of OCT-A images in CNV networks, most lesions undergoing treatment demonstrate vascular remodeling, consisting of shrinkage of fine peripheral vessels, arteriogenesis and maturation of the remaining vessels.[19,32] Therefore, increased branching after arteriogenesis translates into a higher FD. Accordingly, our results may reflect different levels of vascular remodeling, being active CNV more commonly associated with lower branching complexity than inactive CNV. We suggest that further studies test this hypothesis by employing a longitudinal design.

LAC is a measure of vessel nonuniformity where higher values reflect heterogeneity and lower values reflect a more homogenous vascular structure. As previously observed by OCTA imaging, the microvascular features of CNV membranes undergo a transformation when treated with anti-VEGF agents: from an homogeneous, tiny branching network to an heterogeneous dead tree appearance.[6] This qualitative observation translates into higher lacunarity within the CNV lesion after anti-VEGF treatment. Our results showed that the differences in lacunarity between active and remission nAMD groups were not significant. As shown by previous studies lacunarity is high in quiescent lesions. Therefore it is reasonable that lacunarity values were not different between the groups of our study.[6,33] These findings are consistent with the results of Roberts *et al*. (2017) who found non-significant differences in lacunarity between good and poor responders to anti-VEGF therapy.[11]

Our study limitations include a small sample size and a retrospective, cross-sectional design. Low-quality image acquisition decreases the likelihood of detecting small or poorly perfused CNVs and heavily vascularized PEDs. Repeatability of fractal dimension analysis is susceptible to image quality. In low quality images, vessel segments may appear discontinuous which may significantly affect the assessment of branching complexity. We excluded CNV blood flow images with poor imaging quality to overcome this limitation. However, such exclusion could have created a selection bias in favor of CNVs with an hyperintense OCTA signal and patients with clear media and a better ability to fixate. We have also noticed that the duration of the disease was shorter in patients with active disease and that these patients received more IVIs as compared with patients in the remission group. This difference was a consequence of a consecutive inclusion of patients and may have introduced some bias towards inclusion of more patients who are poor responders in group 1. Further studies will be needed to characterize OCT-A parameters according to the response to anti-VEGF, which was not the aim of this study.

Finally, we must also be aware of the limitations of interpreting a three-dimensional structure using two-dimensional image projection. In our opinion, volumetric evaluation and three-dimensional fractal analysis will eventually give the best results in future studies.

In this study, we defined active nAMD as the observation of indirect signs of vascular leakage on structural OCT. However, it is well known that the amount of leakage may not completely relate to the occurrence of fluid in or under the retina. As previously pointed out by Spaide[2], there are several factors related to fluid accumulation which are not easily measurable, including the function of the retinal pigment epithelium, the health of the choroid, and the presence of an intact external limiting membrane. We must also acknowledge that blood flow appearance will not explain all the variability inherent in CNV complexity. The discussed OCT-A biomarkers may add helpful and measurable information to structural OCT interpretation and may help the clinician in the decision-making process of scheduling visits and intravitreal treatments.

## Conclusions

Our study shows that there are measurable characteristics of blood flow on OCT-A that are associated with exudative signs on structural OCT—the study model (Area+FD) demonstrate a good fit to the data. The relationship between FD on OCT-A and exudative signs on structural OCT suggests the development of personalized treatment and follow-up cycles.

By further studying these new biomarkers, OCT-A may become a valuable technique in assessing how CNVs respond to anti-VEGF agents and enable the clinician to make personalized treatment decisions. Meanwhile, we recommend larger, prospective longitudinal studies to evaluate neovascular networks with fractal analysis over the course of their treatment.
